# Influence of cusp reduction and fiber reinforcement on cusp deflection and fracture strength of restored endodontically treated molar teeth

**DOI:** 10.1007/s00784-025-06297-2

**Published:** 2025-04-04

**Authors:** Merve Aksoy Yüksek, Cemile Kedici Alp, Ceyda Sarı, Oya Bala

**Affiliations:** 1 Department of Restorative Dentistry, Faculty of Dentistry, Van Yüzüncü Yıl University, Van, Türkiye; 2 Department of Restorative Dentistry, Faculty of Dentistry, Gazi University, Ankara, Türkiye; 3 Department of Restorative Dentistry, Faculty of Dentistry, İstanbul Medipol University, Istanbul, Türkiye

**Keywords:** Root canal therapy, Fiber, Dental cavity preparations, Fracture strength

## Abstract

**Objective:**

This study aimed to investigate the influence of various cusp reduction amounts, localization, and the use of coronal polyethylene fiber on cusp deflection in endodontically treated molar teeth.

**Material and methods:**

The 120 intact molar teeth observed in the study were divided into 12 groups, and standard MOD cavities were prepared after the root canal treatment was completed. Groups 1 and 2 were determined as positive and negative control groups, respectively. Two cusp reductions were applied to Groups 3, 4, 5, and 6, and functional cusps reduction were applied to Groups 7, 8, 9, and 10. The cusp reductions were either 1.5 mm or 3 mm. After preparation, Groups 3, 5, 7, 9, and 11 were restored with Filtek One Bulk-Fill, while Groups 4, 6, 8, 10, and 12 were restored with Filtek One Bulk-Fill + Ribbond. As the teeth were restored, the amount of cusp deflection was measured with a twin channel deflection gauge device. The fracture strength of the teeth was measured using a universal test machine. The experimental results were statistically evaluated with two-way and one-way ANOVA tests.

**Results:**

The reinforcement of the coronal structure with fiber reduced cusp deflection and increased fracture strength. The statistically significantly less cusp deflection was obtained in the groups with the same cavity design of having fiber placed than in the groups without fiber placed (*p* < 0.001). The statistically significantly lower fracture strength was obtained in Group 11 than all the reduced groups except Groups 5 and 7 (*p* < 0.05). The statistically lower fracture strength was obtained in Group 12, than in Group 4 (*p* < 0.05). Experimental groups with cusp reduction showed less cusp deflection (*p* < 0.001) and higher fracture strength (*p* < 0.05) than experimental groups without cusp reduction.

**Conclusions:**

Fiber application and cusp reduction may have positive effects in terms of reducing cusp deflection and increasing fracture strength in direct restoration cases involving excessive substance loss in endodontically treated teeth.

**Clinical relevance:**

Incorporating cusp reduction and coronal fiber applications in treatment plans for endodontically treated teeth ensures that restorations are both functional and durable.

## Introduction

Endodontic treatment alters the mechanical and physical properties of teeth by causing changes in tooth structure [1–3]. Endodontically treated teeth may exhibit an excessive loss of substance in the tooth structure due to caries, trauma, and cavity preparation. The extent of material loss in the tooth structure reduces the fracture strength of the tooth. Reeh et al. reported that endodontic treatment reduces tooth hardness by only 5%, and the most important reason for the decrease in fracture strength is the amount of material loss in the tooth [4–6].

The survival rate of endodontically treated teeth depends on high-quality restoration and endodontic treatment. According to one study, coronal restoration has a greater effect on the clinical success of endodontically treated teeth than endodontic treatment [7]. Determining the most appropriate restoration for endodontically treated teeth depends on the restoration’s to reconstruct the strength, form, function, and aesthetics of natural teeth [8]. Therefore, planning the restoration of endodontically treated teeth is important for long-term teeth health.

Several studies have recommended adhesive restorations for these teeth [4, 5]. Several treatment alternatives have been developed, such as resin composite restorations, post applications, fiber-reinforced resin composite restorations, cusp coverage restorations, crown restorations, endo-crown restorations, inlay restorations, and onlay restorations.

Cusp coverage is recommended when the cavity isthmus width is greater than two-thirds of the intercusp distance or half of the buccolingual distance [9–11]. Cusp coverage restorations for endodontically treated teeth are considered a more conservative treatment approach than crown restorations; in addition, cusp reduction has been reported to increase fracture strength and prolong the clinical life of teeth [10, 12, 13]. Although there are many studies on the amount and location of cusp reduction, there is no consensus about which cusp reduction design should be used in which situation [12, 13].

Using fiber-reinforced composites or fiber reinforcement in the coronal structure of endodontically treated teeth may increase the fracture strength of restorations. A recent study reported that fiber-reinforced resin composites can serve as alternatives to crown restorations (in terms of fracture resistance) in the restoration of endodontically treated teeth [14].

Several studies have investigated the effect of different resin composites on cusp deflection in resin composite restorations [15, 16], while studies focusing on the effectiveness of fiber-reinforced resin composite restorations on cusp deflection remain limited [17]. Thus, the effect of cusp reduction on cusp deflection has not been thoroughly examined in the literature.

The aim of this study was to investigate the effect of two different reduction amounts (1.5 mm and 3 mm), the localization of the reduction design (functional cusp reduction or two cusp reductions), and coronal polyethylene fiber application on cusp deflection and fracture strength in endodontically treated molars. The first null hypothesis stated that the amount of cusp reduction does not affect cusp deflection and fracture strength. The second null hypothesis was that fiber application with cusp reduction does not affect cusp deflection and fracture strength.

## Material and methods

This research was conducted according to the stipulations of the Helsinki Declaration (2000) and was previously approved by the Gazi University Faculty of Dentistry Clinical Research Ethics Committee under protocol number 2021.06/2. The sample size was determined using G*Power software (version 3.0.10 Franz Faul, Kiel University, Germany). The effect size was 0.617, and the type 1 error (α) was 0.05. The analysis power was 0.90, resulting in a minimum of 10 teeth per tested group. A total of 120 intact molar teeth extracted for periodontal reasons without caries, restorations, cracks, or defects from individuals between the ages of 18–40 were included in the study. A 0.1% thymol solution was used to store the teeth until use. A digital caliper (Insize 1112–150, Insize Inc., USA) was used to measure the buccolingual and mesiodistal dimensions of the teeth. After the teeth were evaluated with a one-way ANOVA test according to their sizes so that there was no statistical difference between the groups (*p* > 0.05), 12 groups were formed with 10 teeth in each group (*n* = 10) (Table 1).

**Table 1:** Mean ± SD values (in mm) of MD and BL dimensions of the teeth

Groups	MD dimentions	BL dimentions
Group 1 (positive control group)	10.25 ± 0.92 *	10.54 ± 0.77 *
Group 2 (negative control group)	10.29 ± 1.31 *	10.47 ± 0.99 *
Group 3	10.29 ± 0.98 *	10.62 ± 0.78 *
Group 4	10.29 ± 1.00 *	10.40 ± 0.82 *
Group 5	10.25 ± 0.76 *	10.61 ± 0.93 *
Group 6	10.25 ± 0.94 *	10.42 ± 0.73 *
Group 7	10.28 ± 1.03 *	10.57 ± 0.67 *
Group 8	10.25 ± 0.77 *	10.70 ± 0.62 *
Group 9	10.29 ± 0.78 *	10.64 ± 0.7 *
Group 10	10.26 ± 0.48 *	10.58 ± 0.89 *
Group 11	10.29 ± 0.71 *	10.70 ± 0.67 *
Group 12	10.28 ± 0.56 *	10.41 ± 0.73 *
*p*-value	***p* = 0.986**	***p* > 0.05**

*Values indicate homogeneous subsets within each dimension (*p* > 0.05), *MD* mesio-distal; *BL* bucco-lingual

Before the tooth cavities were prepared, the distance between the cusps was measured using a digital caliper. Standardized Class II MOD preparations were designed with a buccopalatal width equal to 2/3 of the intercuspal width, and the proximal boxes were designed to 2/3 the width at the tooth equator [18, 19]. The gingival step was designed to be 1 mm higher than the cementoenamel junction. Throughout the preparation process, the buccal and lingual walls were carefully kept parallel. The dimensions of the MOD cavity and the thickness of the remaining cavity walls were checked throughout the preparation using digital calipers and periodontal probes [20].

Ni–Ti rotary instruments (final size F3; Protaper, Dentsply Maillefer, Switzerland) were used with the crown-down technique for root canal treatment. The root canals were irrigated with physiological saline and 2.5% sodium hypochlorite and then dried with paper points (Diadent Group International, Almere, Netherlands). The prepared root canals were filled with root canal filling paste (AH26 sealer, Dentsply Detrey, Konstanz, Germany) and gutta-percha using a single cone (Dentsply Maileffer, Ballaigues, Switzerland). The canal openings were sealed with resin-modified glass ionomer cement (Ketac Cem Plus, 3 M ESPE, USA). Resin-modified glass ionomer cement was placed in the pulp chamber and the cavity depth was standardized as 4.5 mm.

To simulate the periodontal tissue space, the roots were coated with a thin film of pink wax 2 mm below the cementoenamel junction. The teeth were then fixed with acrylic resins 2 mm below the cementoenamel junction into round plastic molds with a length of 30 mm and a diameter of 25 mm.

Reference points were formed on the buccal and lingual surfaces of the teeth that would come into contact with the twin channel deflection gauge probes (BPX44, TESA, Hexagon, Spain). An automatic matrix band (Dispodent, Chennai, India) compatible with the Supermat Matrix System (Hawe Neos Dental, Switzerland) was applied to all teeth in the other group, except those used in the control groups. Before the matrix band was placed, the window opened to the buccal and lingual walls of the matrix bands.

The cusp reduction design was parallel to the occlusal plane without bevels. Before the reduction began, a marking was made on the surface to be reduced in an area close to the cemento-gingival margin. The amount of reduction was continuously checked via regular measurement with a digital caliper. After cavity preparations and endodontic treatments, the cusps in the cusp reduction groups (Groups 3–10) were reduced with combinations of different thicknesses (1.5 mm and 3 mm) and designs (both buccal-lingual and only functional). In the groups where the 1.5 mm reduction was prepared, the resin composite was placed to a height of 1.5 mm, and the restoration was completed. In the groups where the 3 mm reduction was prepared, the resin composite was placed to a height of 3 mm, and the restoration was completed. Reduced cusp amount was marked on the automatic matrix band, and the occlusal morphology was formed using a silicone index [21]. In this way, the occlusal build-up was standardized.

**Fig. 1:**
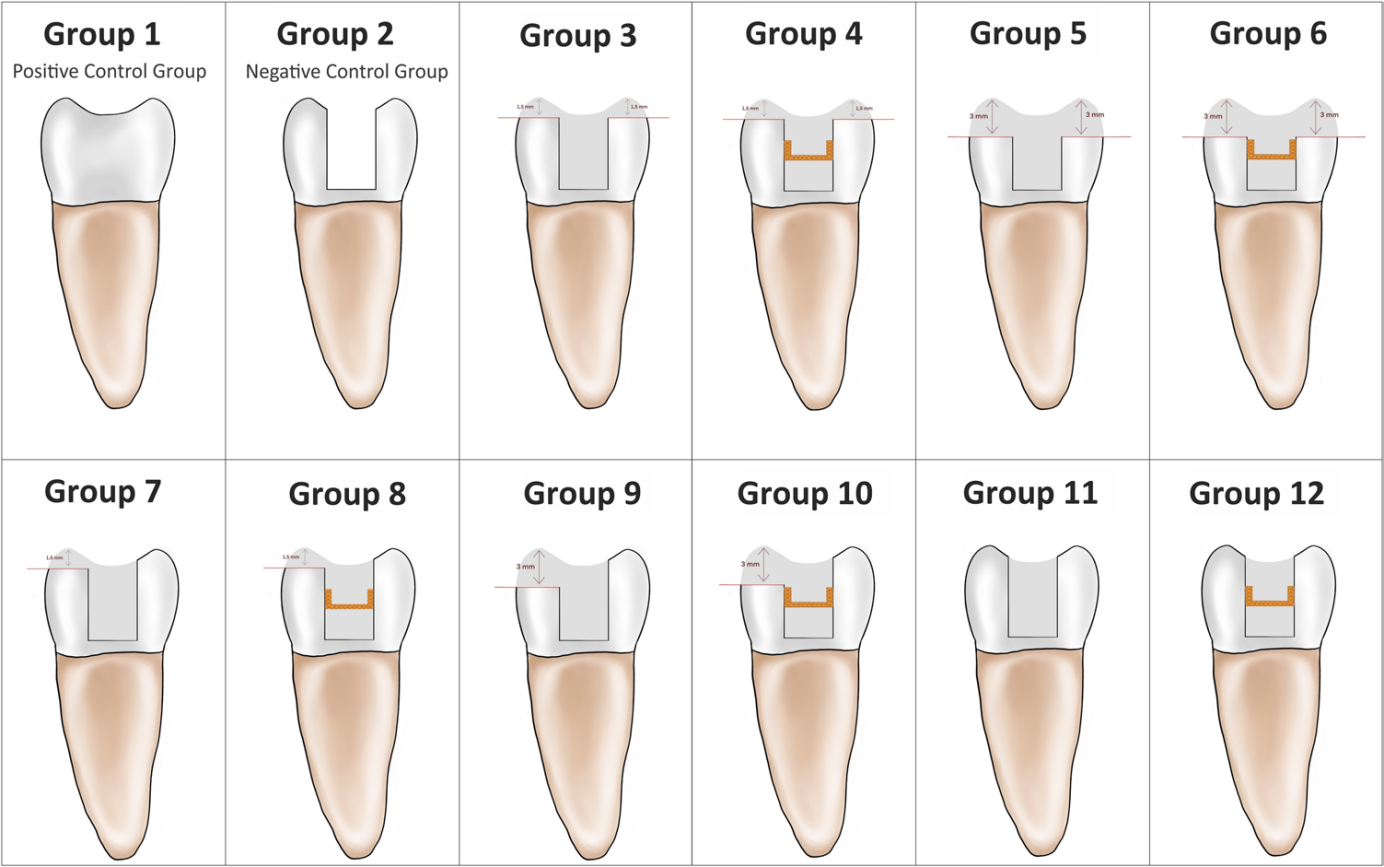
Schematic design of the groups

The groups were designed as follows (Fig. 1):

- Group 1: No treatment was applied to the sound molars (positive control group).
- Group 2: Standard MOD cavities were prepared. No restoration was applied (negative control group).
- Group 3: 1.5 mm buccal and lingual cusp reductions were prepared to the teeth. The teeth were subjected to acid etching using 37% orthophosphoric acid (Scotchbond Universal Etchant – 3 M ESPE) for 30 s for enamel and 15 s for dentin. Then, the teeth were rinsed with water and dried gently. An adhesive (Scotchbond Universal Adhesive, 3 M ESPE, St. Paul, MN, USA) was applied to the cavity for 20 s; this was then rubbed with a microbrush followed by a gentle application of air. The adhesive was then polymerized using an LED light device (BA Optima 10 LED LCUs, BA International, UK) for 20 s. The power density of the light (1200mW/cm^2^) was checked every 5 specimens with a digital dental radiometer (Hilux Light Meter, Benlioğlu, Türkiye). Filtek One Bulk-Fill (3 M, St. Paul, MN, USA) resin composite was applied in the cavity in 4 mm layers. Each layer was polymerized for 20 s using an LED light device.
- Group 4: 1.5 mm buccal and lingual cusp reductions were prepared for the teeth. The same adhesive procedure used for Group 3 was applied in this group. Polyethylene fiber (Ribbond Inc., Seattle, WA, USA) was adapted buccolingually to the middle third of the tooth with Filtek One Bulk-Fill to a thickness of 4 mm, and they were polymerized together for 20 s using an LED light device.
- Group 5: 3 mm buccal and lingual cusp reductions were prepared for the teeth. The same adhesive procedure used for Group 3 was applied in this group. Filtek One Bulk-Fill resin composite was applied in the cavity in 4 mm layers, and each layer was polymerized for 20 s using an LED light device.
- Group 6: 3 mm buccal and lingual cusp reductions were prepared for the teeth. The same adhesive procedure used for Group 3 was applied in this group. Fiber and composite applications were performed in the same as in Group 4.
- Group 7: 1.5 mm functional cusp reduction was prepared for the teeth. The same adhesive procedure used for Group 3 was applied in this group. Filtek One Bulk-Fill resin composite was applied in the cavities in 4 mm layers. Each layer was polymerized for 20 s using an LED light device.
- Group 8: 1.5 mm functional cusp reduction was prepared for the teeth. The same adhesive procedure used for Group 3 was applied in this group. Fiber and composite applications were performed in the same as in Group 4.
- Group 9: 3 mm functional cusp reduction was prepared for the teeth. The same adhesive procedure used for Group 3 was applied in this group. Filtek One Bulk-Fill resin composite was applied in the cavity in 4 mm layers. Each layer was polymerized for 20 s using an LED light device.
- Group 10: 3 mm functional cusp reduction was prepared for the teeth. The same adhesive procedure used for Group 3 was applied in this group. Fiber and composite applications were performed in the same as in Group 4.
- Group 11: No modifications were made to the standard MOD cavities prepared. The adhesive procedure was applied as described in Group 3. Filtek One Bulk-Fill resin composite was applied in the cavity in 4 mm layers. Each layer was polymerized for 20 s using an LED light device.
- Group 12: No modifications were made to the standard MOD cavities prepared. The adhesive procedure was applied as described in Group 3. Fiber and composite applications were performed in the same as in Group 4.

Cusp lengths were measured with a digital micrometer. If the cusp lengths differed, the shortest cusp was used as a reference for cusp reduction. Samples with a difference of more than 0.5 mm between cusp lengths were not included in the study.

The polyethylene fiber used in the study was 3 mm long, soaked with adhesive, and placed buccolingually in the middle third part of the tooth in the form of two strips [22]. The fiber band was only limited to the inside of the cavity. The manufacturer’s instructions for the use of resin composites were followed.

### Measurement of cuspal deflection

**Fig. 2:**
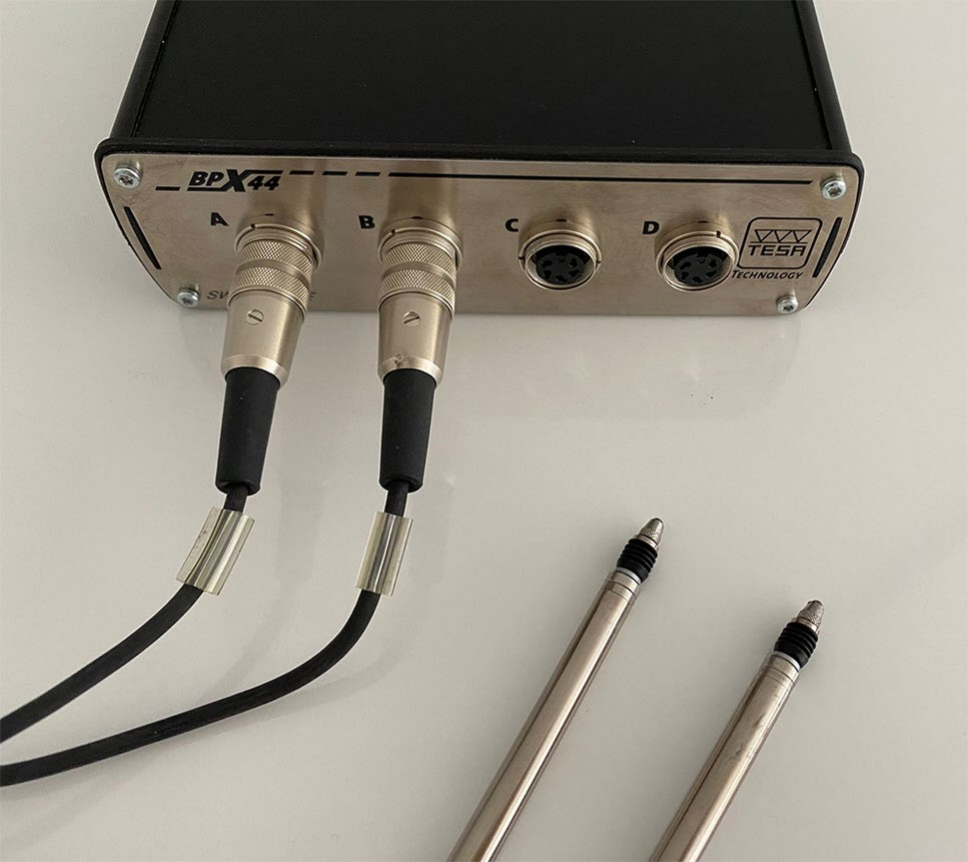
Twin channel deflection gauge, TESA BPX44

**Fig. 3:**
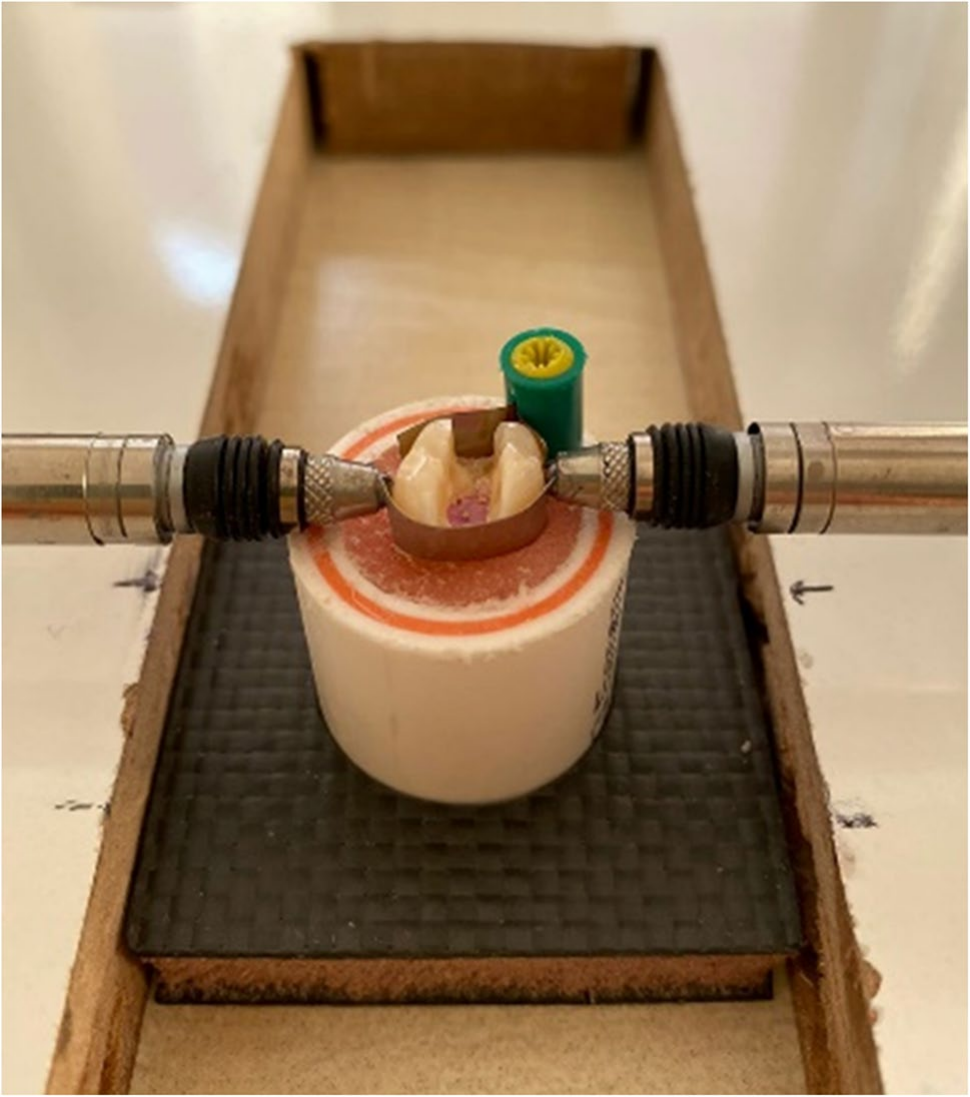
Measurement of cusp deflection

The amount of cusp deflection that may occur during polymerization was measured with a twin-channel deflection gauge probe (TESA BPX44) (Fig. 2) and Tesa Interface Software. Before each measurement, the device was calibrated using a master block. The teeth were placed in the device based on the reference points that the probes would make contact on the buccal and lingual surfaces of the teeth (Fig. 3). The distance between the cusps was measured before the restoration and recorded as the initial measurement. Cusp deflection in teeth was measured at different periods: initially, immediately after the restoration was completed, 30 s later, 3 min later, 4 min later, and 5 min later.

### Measurement of fracture strength

**Fig. 4:**
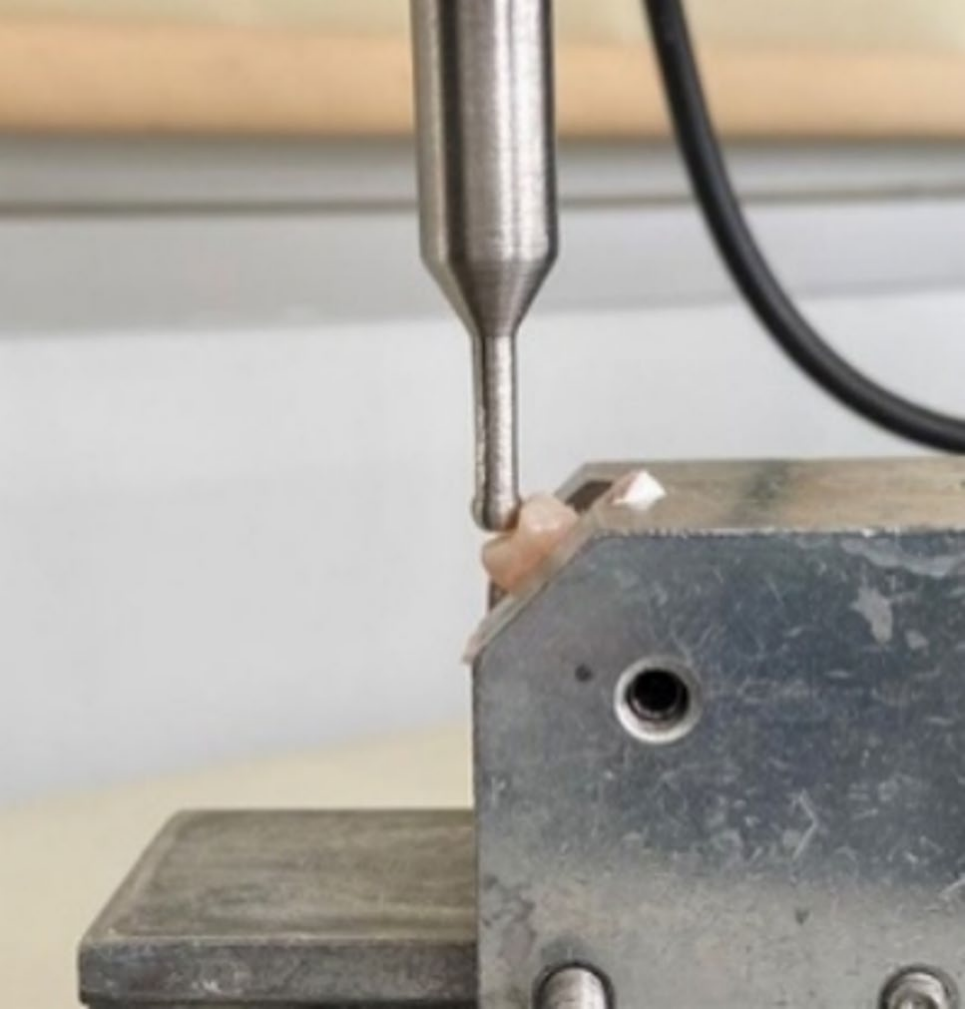
Measurement of fracture strength

After measuring the amount of cusp deflection of the teeth, the samples were kept in an incubator for 24 h at 37 °C to complete the post-polymerization. The fracture strength of the teeth was measured with a universal test machine (Shimadzu, Tokyo, Japan) (Fig. 4). In order to accurately reflect the forces involved in mastication movements, the restored teeth were placed at an angle of 45° to the device similar to Nezir et al. [23], and a spherical stainless-steel tip was placed in contact with the middle section of both functional cusps of the teeth. The load was applied to the central fossa at 1 mm/min.

The restored teeth were placed at an angle of 45° to the device. A spherical stainless-steel tip was placed in contact with the functional cusps of the teeth, and the load was applied at 1 mm/min. To accurately reflect the forces in mastication movements, the central fossa was loaded with the occlusal load at 45**°** to the long axis of the tooth [23, 24].

The fracture types were categorized as follows: fractures with fracture lines extending up to 1 mm below the cementoenamel junction (“restorable fractures”) and fractures more than 1 mm below the cementoenamel junction (“unrestorable fractures”) [25].

### Statistical analysis

Data were analyzed using the IBM SPSS Statistics 25.0 (IBM Corporation, Armonk, NY, USA) program. A two-way ANOVA test was conducted to determine whether there was a statistically significant difference between the groups in terms of fracture strength. When the results of the two-way ANOVA test were found to be significant, the group(s) causing the said difference were investigated using the Dunn-Bonferroni test. A one-way ANOVA test was used to determine whether the mean change in cusp movements between any two follow-up measures showed a statistically significant difference between the groups. When the results of the one-way analysis of variance were found to be significant, the group(s) causing the said difference were determined using the post-hoc Tukey HSD test. The statistical significance level was set at *p* < 0.05.

## Results

### Evaluation of the amount of cusp deflection

The means and standard deviations of the cusp deflection data are shown in Table 2 and Table 3. The highest cusp deflection values among the follow-up times in all groups were obtained between the initial measurement and immediately after polymerization. The total cusp deflection between the initial measurement and the measurement after five minutes was analyzed among the groups, with Group 11 having the highest cusp deflection (83.52). Cusp reduction was not prepared in this group; the teeth were restored with resin composite and without fiber application. An evaluation of the reduction groups revealed that Group 3 (47.04), in which both cusps were reduced by 1.5 mm, had more cusp deflection than all other reduction experimental groups (*p* < 0.001). The lowest cusp deflection was obtained in Group 6 (10.59), in which both cusps were reduced by 3 mm and fiber was placed, and in Group 10 (10.94), in which the functional cusp was reduced by 3 mm and fiber was placed. No statistically significant differences were identified among Groups 6 to 10 (*p* > 0.0033).

**Table 2:** The amount of cusp deflection change in the experimental groups in successive time periods

Groups	Initial—after immediately	After immediately—after 30 s	After 30 s—after 3 min	After 3 min—after 4 min	After 4 min – after 5 min
Group 3	45.06 ± 5.41^bcdefghij^	0.87 ± 0.57	0.84 ± 0.58	0.18 ± 0.12	0.08 ± 0.04^e^
Group 4	17.32 ± 3.95^abcde^	0.95 ± 0.54	0.80 ± 0.37	0.21 ± 0.18	0.11 ± 0.08^e^
Group 5	22.50 ± 2.49^abcde^	1.02 ± 0.46	0.84 ± 0.25	0.16 ± 0.07	0.09 ± 0.04^e^
Group 6	8.46 ± 4.95^acdfg^	0.85 ± 0.48	0.63 ± 0.34	0.17 ± 0.11	0.47 ± 0.40^abcdfhi^
Group 7	21.34 ± 3.14^abcd^	0.80 ± 0.46	0.79 ± 0.46	0.17 ± 0.12	0.16 ± 0.07^f^
Group 8	15.43 ± 3.52^acd^	1.11 ± 0.39	1.10 ± 0.35	0.20 ± 0.15	0.18 ± 0.10
Group 9	21.14 ± 2.48^abcd^	0.96 ± 0.43	0.58 ± 0.29	0.13 ± 0.09	0.18 ± 0.25
Group 10	8.89 ± 6.71^acd^	0.98 ± 0.57	0.76 ± 0.36	0.16 ± 0.09	0.17 ± 0.11^e^
Group 11	81.40 ± 5.34^ac^	1.19 ± 1.37	0.59 ± 0.73	0.22 ± 0.15	0.12 ± 0.09^e^
Group 12	66.82 ± 4.7^abcdefghij^	0.88 ± 0.82	0.98 ± 0.55	0.17 ± 0.10	0.12 ± 0.07^e^
*p*-value †	** < 0.001**	0.968	0.217	0.867	** < 0.001**

†One way ANOVA analysis. Results were considered statistically significant for *p* < 0.0033, according to Bonferroni Correction. a: The difference between them and Group 3 is statistically significant (*p* < 0.001), b: The difference between them and group 10 is statistically significant (*p* < 0.001), c: The difference between them and Group 11 is statistically significant (*p* < 0.001), d: The difference between them and group 12 is statistically significant (*p* < 0.001), e: The difference between them and group 6 is statistically significant (*p* < 0.001), f: The difference between them and group 7 is statistically significant (*p* < 0.001), g: The difference between them and group 9 is statistically significant (*p* < 0.001), h: The difference between them and group 4 is statistically significant (*p* < 0.001), i: The difference between them and group 5 is statistically significant (*p* < 0.001), j: The difference between them and group 8 is statistically significant (*p* < 0.001)

**Table 3:** The total amount of cusp deflection change in the experimental groups

Groups	Initial—After 5 min
Group 3	47.04 ± 5.49^bcdefghij^
Group 4	19.40 ± 3.82^abcde^
Group 5	24.62 ± 2.35^abcde^
Group 6	10.59 ± 5.15^acdfghi^
Group 7	23.26 ± 3.18^abcde^
Group 8	18.02 ± 3.40^acd^
Group 9	22.98 ± 3.05^abcde^
Group 10	10.94 ± 6.61^acdfghij^
Group 11	83.52 ± 5.40^abdefghij^
Group 12	68.97 ± 4.95^abcefghij^
p-değeri †	** < 0.001**

†One way ANOVA analysis. Results were considered statistically significant for *p* < 0.0033 according to Bonferroni Correction. a: The difference between them and group 3 is statistically significant (*p* < 0.001), b: The difference between them and group 10 is statistically significant (*p* < 0.001), c: The difference between them and group 11 is statistically significant (*p* < 0.001), d: The difference between them and group 12 is statistically significant (*p* < 0.001), e: The difference between them and group 6 is statistically significant (*p* < 0.001), f: The difference between them and group 7 is statistically significant (*p* < 0.001), g: The difference between them and group 9 is statistically significant (*p* < 0.001), h: The difference between them and group 4 is statistically significant (*p* < 0.001), i: The difference between them and group 5 is statistically significant (*p* < 0.001), j: The difference between them and group 8 is statistically significant (*p* < 0.001).

The analysis of the effect of fiber placement on cusp deflection revealed that statistically significantly less cusp deflection was obtained in the groups with the same cavity design of having fiber placed than in the groups without fiber placed except Groups 7 (23.26 ± 3.18) and 8 (18.02 ± 3.40) (*p* < 0.001).

As to the effect of cusp reduction on cusp deflection is evaluated, statistically lower cusp deflection was obtained in Group 7 (23.26), in which the functional cusp was reduced by 1.5 mm, than in Group 3 (47.04), in which both cusps were reduced by 1.5 mm (*p* < 0.001). Statistically lower cusp deflection was obtained in Group 5 (24.62), in which both cusps were reduced by 3 mm, and in Group 3 (47.04), in which both cusps were reduced by 1.5 mm (*p* < 0.001). Statistically lower cusp deflection was obtained in Group 6, in which both cusps were reduced by 3 mm and fiber was placed, than in Group 4 (19.40), in which a 1.5 mm reduction was applied to both cusps and fiber was placed (*p* < 0.01).

### Evaluation of fracture strength

**Fig. 5:**
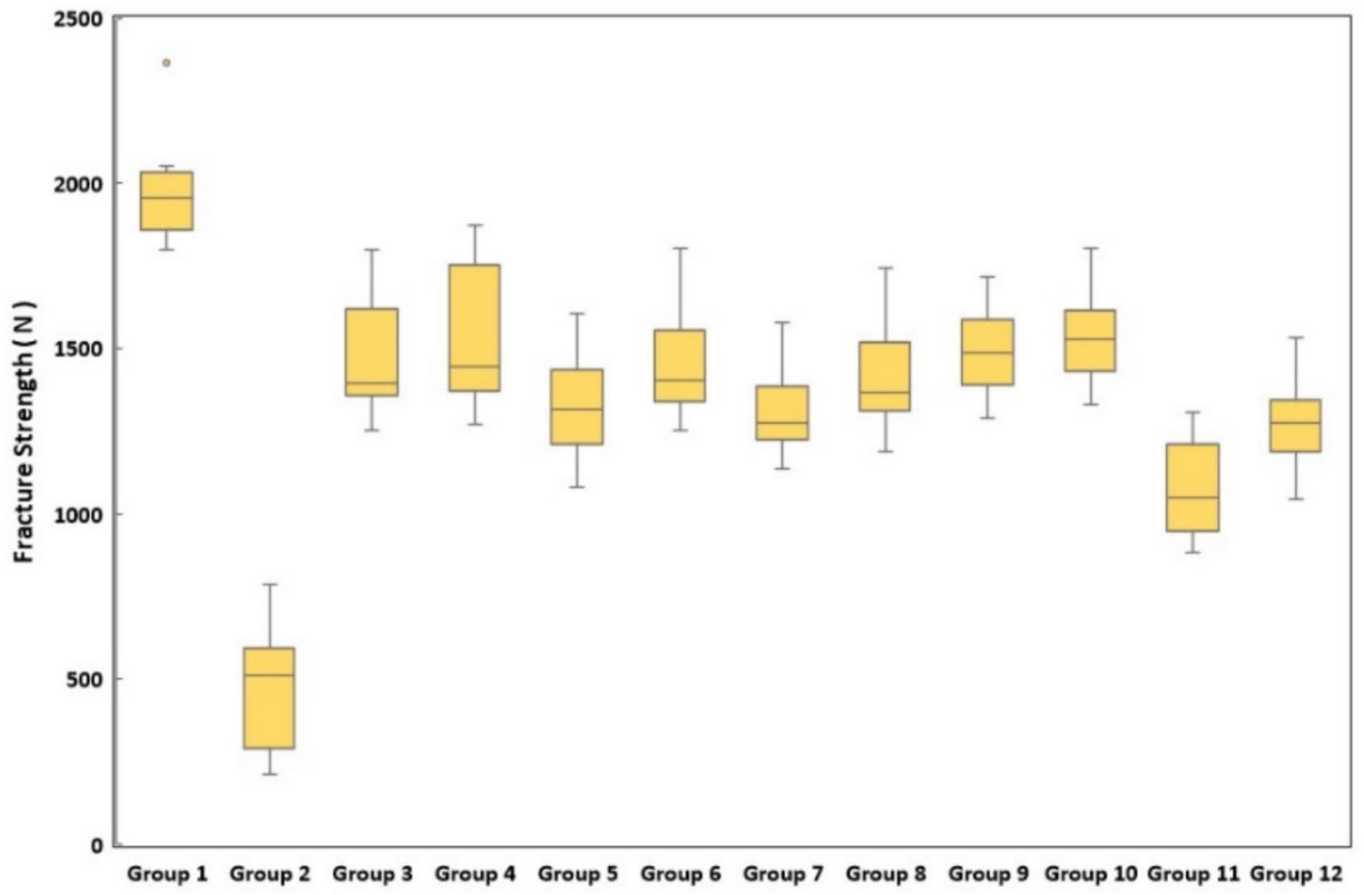
Fracture strength values of the groups

The means and standard deviations of the data obtained from the fracture strength test are shown in Table 4 and Fig. 5.

**Table 4:** Mean fracture strength values and standard deviations of the groups

Experimental Groups	Mean	Standard Deviation
Group 1 (positive control group)	1970.206^b^	165.02
Group 2 (negative control group)	481.429^a^	205.08
Group 3	1466.929^abc^	181.68
Group 4	1544.526^abcd^	225.11
Group 5	1336.223^ab^	167.15
Group 6	1460.371^abc^	178.55
Group 7	1304.264^ab^	140.27
Group 8	1407.277^abc^	177.25
Group 9	1488.422^abc^	172.75
Group 10	1534.243^abc^	144.61
Group 11	1082.468^ab^	154.64
Group 12	1282.321^ab^	143.21
*p*-value	< 0.05	

a: The difference with Group 1 is statistically significant (*p* < 0.05), b: The difference with Group 2 is statistically significant (*p* < 0.05), c: The difference with Group 11 is statistically significant (*p* < 0.05), d: The difference with Group 12 is statistically significant (*p* < 0.05)

An analysis of the fracture strengths of the groups indicated that the highest fracture strength was observed in the positive control group (1970.206 MPa), while the negative control group had the lowest observed fracture strength (481.429 MPa). The experimental groups showed statistically significantly lower fracture strength than the positive control group (*p* < 0.05). In addition, the experimental groups exhibited statistically significantly higher fracture strength than the negative control group (*p* < 0.05).

Statistically significantly lower fracture strength was obtained in Group 11 (1082.468), which did not have any reductions applied and did not have any fiber placed, than all the reduced groups except Groups 5 (1336.223) and 7 (1304.264) (*p* < 0.05). Statistically lower fracture strength was obtained in Group 12 (1282.321), in which fiber was placed without reduction, than in Group 4 (1544.526), in which 1.5 mm reduction was applied to both cusps and fiber was placed (*p* < 0.05). No statistically significant differences were identified among the other groups (*p* > 0.05).

The cusp reduction evaluation revealed that the highest fracture strength was obtained when both cusps were reduced by 1.5 mm and fiber was applied (Group 4), although this finding was not statistically significant.

According to the evaluation of the effect of fiber application on fracture strength, additional fracture strength was obtained in the fiber-placed groups than in the non-fiber-placed groups with the same cavity design, although this was not a statistically significant finding.

**Fig. 6:**
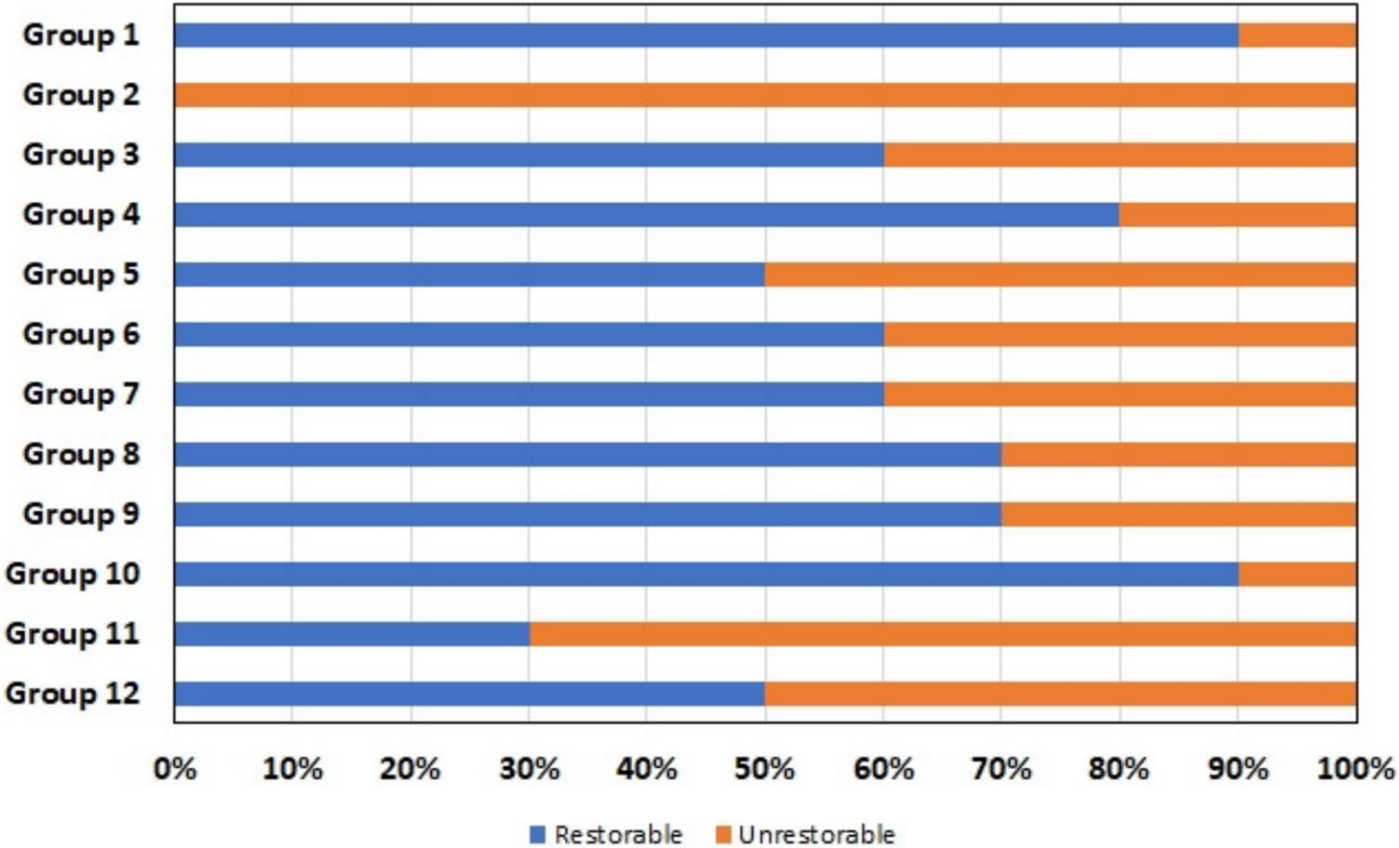
Distribution of fracture types of the groups

Restorable fracture rates were highest in the positive control group and Group 10, which had 3 mm functional cusp reduction and fiber application, while the lowest rates were observed in the negative control group (Fig. 6).

## Discussion

In vitro studies examining the effect of restoration types on restoration life may guide future restorative treatment procedures. In this study, the effect of modifying the restorations by cusp reduction and polyethylene fiber application on cuspal deflection and fracture strength was investigated.

The results of this study show that cusp reduction increased the fracture strength and decreased the cusp deflection values of the restorations. Thus, the first null hypothesis was rejected. Furthermore, the application of fiber was found to increases the fracture strength of restorations compared to the application of only bulk-fill resin composite. The effect of Ribbond on cusp deflection was evaluated, and fiber application was found to significantly reduce cusp deflection. Therefore, the second null hypothesis was rejected.

The fracture strength test is a method that is frequently used to measure the strength of teeth against forces, as it is easily applicable and repeatable [12, 17, 26–28]. The twin channel deflection measuring gauge is a relatively easy device for the operator to use and stabilize. In this study, a fracture strength test and twin channel deflection measuring gauge were used to evaluate restorations (Fig. 2).

Indirect restorations for endodontically treated teeth constitute treatment options that have recently increased in popularity. The literature contains mixed results regarding the clinical performance of direct and indirect composite restorations for posterior teeth. Abbas et al. investigated direct resin composite restorations, overlays, conventional crowns, and endocrowns, finding that the highest fracture resistance was observed in conventional crowns and that the fracture resistance was identified in direct resin composite restorations [29]. Conversely, de Kuijper et al. reported that direct resin restorations obtained similar fracture strength to lithium disilicate crowns and endocrowns [25]. In a study by Torabzadeh et al., it was reported that direct resin restorations with cusp coverage obtained higher fracture strength than indirect onlay resin restorations, although this finding was deemed not statistically significant [30]. The available evidence is inconclusive due to the high risk of bias in many studies and the lack of long-term randomized controlled trials [31]. Factors such as the amount of remaining tooth tissue, socioeconomic conditions, and restorative indications may influence the choice between direct and indirect techniques [31, 32]. Should direct restorations be preferred in root canal–treated teeth, resin composite restorations can often be applied with various modifications.

Bulk-fill resin composites may facilitate the placement of dental fibers used to strengthen coronal restorations. In this study, Filtek One Bulk-Fill resin composite was used to restore root canal–treated teeth. After placing the bulk-fill resin composite in the middle third of the cavity, the Ribbond was placed, and the upper part of the restoration was completed in a single layer. After placing the Ribbond, applying the bulk-fill resin composite in the top layer may have stabilized the Ribbond. The leno-weaving characteristic of Ribbond allows the forces to be distributed over a wider area, thereby increasing the stability of the fiber [26].

The dentin was removed until the reaching the pulp chamber roof. Endodontic access cavities were prepared in the teeth. Different dimension of pulp chamber in each tooth are one of the difficulties in the restoration of endodontically treated teeth [33]. In order to standardize the cavity depth, the pulp chamber was filled to the 4.5 mm depth with resin-modified glass ionomer cement for each tooth. The resin composite was placed up to the middle of the cavity depth for each tooth. Then, the Ribbond was placed over the composite layer. The upper half was restored again with a resin composite.

There are many types of fiber placement, such as gingival, middle third, occlusal, transcoronal splinting, and circumferentially [23, 34]. In this study, the effect of Ribbond placement in the middle third was investigated.

In our study, lower cusp deflection values were obtained in the groups using Ribbond than in the groups without Ribbond with the same cavity design. Fiber placement may positively affect decreasing cuspal deflection in endodontically treated teeth with high material loss, independent of reduction. In a study investigating the effects of fiber mesh use on cusp deflection in the literature, it was reported the use of a polyethylene fiber mesh reduces cusp deflection. [17]. This is attributed to the leno-weaving property of Ribbond, a polyethylene fiber mesh, which allows for better stress distribution within the restoration. To the best of our knowledge, no article has investigated the effect of cusp reduction on cusp deflection in the dental literature. In this study, the amount and location of cusp reduction, Ribbond placement, and its effect on cusp deflection were evaluated. When Ribbond is used, the amount of cusp deflection decreases, regardless of the reduction and its amount. This result was similar to that of Akman et al. [17], who reported that placing Ribbond in the cavity in different ways in molar teeth on cusp deflection significantly reduced the amount of cusp deflection, regardless of the placement method employed. In this study, akin to the work of Akman et al., two pieces of Ribbond were placed buccolingually on both cusps in molar teeth. In contrast to the study of Akman et al., in endodontically treated molars, Ribbond was placed in the middle third of the teeth, and cusp reduction was added to the cavity design. The amount of cusp deflection was significantly reduced when the fiber was placed in cavities of the same design. Similar to the study of Akman et al., it can be concluded that Ribbond provides a better distribution of stresses in restoration and reduces cusp deflection.

The literature shows that Ribbond placement increases fracture strength. A study by Belli et al. reported that Ribbond application increased the fracture strength in endodontically treated molars with MOD cavities [35]. In a study by Kalburge et al., it was reported that the application of Ribbond in teeth with DO and MOD cavities increased fracture strength [36]. Another study reported that polyethylene fiber reinforcement composite and short-fiber-reinforced composite resin showed greater fracture strength than direct composite resin [37]. In our study, the groups with Ribbond placement exhibited greater fracture strength than the groups without it, regardless of cusp reduction.

Cusp deflection was evaluated at different time periods in various studies. Elsharkasi et al. reported that cusp deflection increased up to a certain period, such as five minutes; then, a slight decrease was observed in the distance between cusps [38]. Studies have reported that the most significant change in the amount of cusp deflection occurs within the first five minutes after the restoration is completed [39, 40]. In this study, the last measurement time was determined as five minutes.

The total cusp deflection was analyzed in the groups. The lowest cusp deflection was seen in Group 6 (10.59) (both cusps had a 3 mm reduction, and fiber was placed) and in Group 10 (10.94) (the functional cusp was made a 3 mm reduction, and fiber was placed). As a result, only a 3 mm reduction in the functional cusp may be sufficient. When the effect of cusp reduction on cusp deflection was analyzed, it was found that functional cusp reduction reduced cusp deflection more. Thus, functional cusp reduction may be sufficient in terms of cusp deflection. Functional cusp reduction reduces cusp deflection, regardless of the amount of cusp reduction. A 3 mm cusp reduction reduces cusp deflection, regardless of the location of the cusp reduction.

The purpose of cusp coverage restorations with cusp reduction is to protect weakened cusps during mastication forces and to increase the fracture strength of the tooth [13]. Although studies are limited on this topic are limited, it has been reported that cusp reduction designs increase the fracture strength of teeth [10, 41, 42]. Chang et al. [43] evaluated the amount of reduction in overlay restorations with their finite element stress analysis studies, noting that the stress distribution in designs of a 1.5 mm reduction or less was similar to the control group without any reduction, and when more reductions were made, the stress distribution changed, and the stress in the dental tissue decreased. In this study, the minimum amount of cusp reduction was 1.5 mm. In the study of Serin Kalay et al. [10], the effects of bevel, horizontal, and anatomic reduction design types in premolar teeth on fracture strength were investigated, and results showed that the 2.5 mm and 3.5 mm cusp reduction results with higher fracture strength than 1.5 mm. In addition, it has been reported that in the design of 2.5 mm cusp reduction, the anatomic reduction design has a higher fracture strength than the horizontal and beveled reduction design. In this study, horizontal cusp reduction was determined to be 1.5 mm and 3 mm. In contrast to the method used by Serin Kalay et al., the effect of functional cusp reduction and the use of Ribbond, which was applied as two fiber strips buccolingually in the middle third in the coronal structure, on cusp deflection were evaluated. In a study by Mondelli et al.[42], it was reported that the fracture strength of the restorations without cusp coverage decreased and that the fracture strength of the restorations with cusp coverage indicated similar values to those of sound teeth. In accordance with the studies of Serin Kalay et al. and Mondelli et al., this study concluded that the effect of reduction on endodontically treated teeth may increase fracture strength.

In contrast to this finding, a study by Soares et al. [44] reported that post placement and cusp reduction reduce the fracture strength. The reason for this divergence may be that fracture strength is potentially affected by many factors, such as the remaining tooth structure and amount, restorative material, and post-placement procedures.

According to our results, even a 3 mm reduction on the functional cusp increases the fracture strength; a 1.5 mm reduction in both cusps may also be sufficient to protect restored teeth.

Fracture types are as important as fracture strength in endodontically treated teeth. The loss of material in the tooth tissue and the materials used for the restoration may affect the fracture type. It has been reported that fiber applications increase the rate of restorable fractures and conserve tooth structure [23, 45–47]. In this study, the most restorable fracture type was observed in Group 10 (which had a 3 mm functional cusp reduction and fiber placement), similar to sound teeth. This was followed by Groups 4 and 8. After the negative control group, the most non-restorable fractures were observed in Group 11, which did not have cusp reduction and did not have fiber placed (Fig. 6). In accordance with the study by Serin Kalay et al. [10], fiber placement increased the rate of restorable fractures in this study. Performing cusp reduction may reduce cusp deflection and create differences in the distribution of forces on restorations; thus, the rate of restorable fractures is higher in restorations involving cusp reduction. One of the limitations of the current study is that the effects of mechanical, thermal fatigue, and aging conditions were not assessed [48]. Future research could investigate the impact of mechanical and thermal fatigue and aging conditions to evaluate long-term outcomes. The other limitations of the study are the use of only molar teeth and the evaluation of only direct restorations in endodontically treated teeth. The study’s results may differ for premolars due to their structural and functional differences, suggesting that comparative studies involving molars could be useful. Additionally, no comparisons were made with indirect restorations in this study. This may warrant further investigation to assess the relative efficacy of direct versus indirect approaches.

## Conclusions

Cavities may be modified with cusp reduction to reduce cusp deflection and increase durability in excessive loss of tooth structure. The application of polyethylene fiber to support the buccal and lingual walls may be recommended.

## Data Availability

No datasets were generated or analysed during the current study.
